# Correction: Intermediate knowledge enhanced the performance of the amide coupling yield prediction model

**DOI:** 10.1039/d6sc90208a

**Published:** 2026-10-27

**Authors:** Chonghuan Zhang, Qianghua Lin, Chenxi Yang, Yaxian Kong, Zhunzhun Yu, Kuangbiao Liao

**Affiliations:** a Guangzhou Municipal and Guangdong Provincial Key Laboratory of Molecular Target & Clinical Pharmacology, The NMPA and State Key Laboratory of Respiratory Disease, School of Pharmaceutical Sciences, Guangzhou Laboratory, Guangzhou Medical University Guangzhou Guangdong 511436 PR China yu_zhunzhun@gzlab.ac.cn; b Guangzhou National Laboratory, Guangzhou International Bio Island No. 9 Xingdaohuanbei Road Guangzhou Guangdong 510005 PR China; c AIChemEco Inc. Guangzhou Guangdong 510005 PR China

## Abstract

Correction for ‘Intermediate knowledge enhanced the performance of the amide coupling yield prediction model’ by Chonghuan Zhang *et al.*, *Chem. Sci.*, 2025, **16**, 11809–11822, https://doi.org/10.1039/d5sc03364k.

Following discussions with the editorial office, the authors have expanded their Conflicts of interest and Acknowledgements sections to ensure that they are as detailed as possible. The full statements are available below.

**Conflicts of interest**

K. L. is the founder and Chief Scientist of AIChemEco Inc. and holds equity in the company. C. Y. and Y. K. are employees of AIChemEco Inc. AIChemEco Inc. provided personnel and in-kind support for this study, including access to its ultra-high-throughput experimentation platform, analytical facilities, software and computational resources, and support for experimental data generation and processing. The other authors declare no competing interests.

**Acknowledgements**

We are grateful for financial support from the National Natural Science Foundation of China, Grant No. 22071249 and 22393892. This work was also supported by the Major Project of Guangzhou National Laboratory, Grant No. GZNL2025C01022, GZNL2024A01005 and GZNL2023A02012.

We also acknowledge in-kind support from AIChemEco Inc., including personnel time and access to its ultra-high-throughput experimentation platform, analytical facilities, software and computational resources, and support for experimental data generation and processing.

The Royal Society of Chemistry apologises for these errors and any consequent inconvenience to authors and readers.

